# Renal iron overload in rats with diabetic nephropathy

**DOI:** 10.14814/phy2.12654

**Published:** 2015-12-23

**Authors:** Jesus H. Dominguez, Yunlong Liu, Katherine. J. Kelly

**Affiliations:** ^1^ Departments of MedicineIndiana University School of MedicineIndianapolisIndiana; ^2^ Roudebush Veterans' Affairs Medical CenterIndianapolisIndiana; ^3^ Medical and Molecular GeneticsIndiana University School of MedicineIndianapolisIndiana

**Keywords:** complement, diabetic nephropathies, ischemia, kidney failure, chronic

## Abstract

Diabetic nephropathy (DN) remains incurable and is the main cause of end‐stage renal disease. We approached the pathophysiology of DN with systems biology, and a comprehensive profile of renal transcripts was obtained with RNA‐Seq in ZS (F_1_ hybrids of Zucker and spontaneously hypertensive heart failure) rats, a model of diabetic nephropathy. We included sham‐operated lean control rats (LS), sham‐operated diabetic (DS), and diabetic rats with induced renal ischemia (DI). Diabetic nephropathy in DI was accelerated by the single episode of renal ischemia. This progressive renal decline was associated with renal iron accumulation, although serum and urinary iron levels were far lower in DI than in LS. Furthermore, obese/diabetic ZS rats have severe dyslipidemia, a condition that has been linked to hepatic iron overload. Hence, we tested and found that the fatty acids oleic acid and palmitate stimulated iron accumulation in renal tubular cells in vitro. Renal mRNAs encoding several key proteins that promote iron accumulation were increased in DI. Moreover, renal mRNAs encoding the antioxidant proteins superoxide dismutase, catalase, and most of the glutathione synthetic system were suppressed, which would magnify the prooxidant effects of renal iron loads. Substantial renal iron loads occur in obese/diabetic rats. We propose that in diabetes, specific renal gene activation is partly responsible for iron accumulation. This state might be further aggravated by lipid‐stimulated iron uptake. We suggest that progressive renal iron overload may further advance renal injury in obese/diabetic ZS rats.

## Introduction

Diabetic nephropathy (DN) remains incurable and still is the main cause for end‐stage renal disease (ESRD) (U.S. Renal Data System, 2013) (Kelly and Dominguez 2010). The pathophysiology of DN is complex, and human studies are necessarily limited in their scope. It is for this reason that animals have been used as surrogates to study disease mechanisms. The ZS (F_1_ hybrids of Zucker and spontaneously hypertensive heart failure) rat is a model of obesity, diabetes, and dyslipidemia with progressive nephropathy (Dominguez et al. 2007), and it is amenable to systematic investigations not possible in humans (Breyer et al. 2005; Temm and Dominguez 2007). As seen in humans, episodes of acute renal injury (AKI) accelerate renal inflammation, apoptosis, fibrosis, and failure in ZS rats (Kelly et al. 2009). We recently showed that systems biology, including deep sequencing with advanced bioinformatics, can be used to study the syndrome of accelerated renal decline (Kelly et al. 2013). This approach showed that obese, diabetic ZS rats with renal ischemia exhibited general activation of the renal complement system along with interacting proinflammatory gene networks (Kelly et al. 2015). The inciting mechanisms of renal C3 activation were likely ischemic and prooxidant, as detailed in earlier work (Kelly et al. 2013, 2015). It is also noteworthy that the source of the renal prooxidant state in ischemia is in part derived from renal iron, as shown in isolated kidneys (de Vries et al. 2006). Therefore, we hypothesized that renal iron overload, observed in diabetes (Rajpathak et al. 2009; Zheng et al. 2011), may also be a component of diabetic nephropathy. We found that rats with diabetes and renal ischemia had higher renal iron loads, even while renal transcripts encoding cellular iron efflux were upregulated.

## Subjects and Methods

### Animal

**Figure 1:**
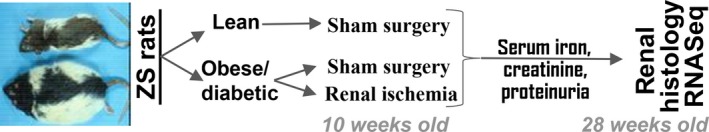
Experimental diagram. Left to right: ZS lean rats were subjected to sham surgery at 10 weeks of age (LS,* n* = 6), whereas obese/diabetic ZS rats were either sham‐operated (DS,* n* = 7) or subjected to renal ischemia for 25 min at 10 weeks of age (DI,* n* = 11). Serum and urine samples were collected and rats terminated at 28 weeks of age. There was a group of 4 DI rats terminated at 36 weeks of age and are included only to demonstrate sustained renal iron accumulation.

The three groups of rats included here, and their core renal transcript networks, including inflammation, have been reported elsewhere (Kelly et al. 2013, 2015) Lean and obese, diabetic male ZS rats (Charles River, Wilmington, MA) were acquired at 8 weeks of age and fed Purina diet #5008. Their body weights were measured and sera were analyzed for creatinine, iron, and total iron binding capacity (TIBC), and urine was analyzed for iron by the clinical laboratory of the Indianapolis VA Hospital. Urine protein was measured as previously described (Kelly et al. 2013). One group of obese/diabetic rats was subjected to bilateral renal ischemia for 25 min at 10 weeks of age as described (DI, or DMisch, *n* = 11) (Kelly et al. 2013). The lean rats (LS, or Lean, *n* = 6) and a second obese/diabetic group (DS, or DMsham, *n* = 7) were subjected to sham surgery. These rats were terminated at 28 weeks of age, their kidneys removed, immediately frozen in liquid nitrogen or fixed in 10% formalin and embedded in paraffin, Figure 1. The frozen sections were used for measurements of renal mRNA (below). There were four additional DI rats that were terminated at age 36 weeks and are only included to demonstrate late and sustained renal iron accumulation.

### Histology

Kidney sections were stained with the Prussian blue protocol. The sections were deparaffinized and then immersed in an acid solution of potassium ferrocyanide, which reacts with renal ferric iron forming a bright blue pigment: Prussian blue, or ferric ferrocyanide. The sections were then counterstained with nuclear fast red. These stained renal sections were also used to measure renal iron by quantitating the blue pixel density of ferric ferrocyanide.

### Immuno(western)blotting

Kidney cortices were homogenized in 25 mmol/L Tris, pH 7.6, 150 mmol/L NaCl, 1% deoxycholate, 1% P‐40, 0.1% SDS, and 2× Halt Protease Inhibitor Cocktail (Thermo Scientific, Rockford, IL), and adjusted to a protein concentration of 2 mg/mL. The homogenates (20 *μ*g) were fractionated by electrophoresis through 16.5% polyacrylamide Tris‐Tricine gels. After electrophoresis, proteins were transferred to a nitrocellulose filter. Blocking was carried out in 1% casein, 1X PBS for 1 h. Incubation with primary antibody diluted in 1X PBS was for 1 h; primary antibody was rabbit anti‐ferritin, heavy chain (1:1000) (Cat # ab81444; Abcam, Cambridge MA) and mouse anti‐actin (1:1000) (clone AC‐40, Sigma‐Aldrich, St. Louis, MO). The filter was then washed in 1X PBS and incubated with secondary antibodies diluted in 1X PBS for 1 h. Secondary antibodies were IRDye 680 goat anti‐rabbit IgG (1:15,000) (Li‐Cor Biosciences, Lincoln, NB) and IRDye 800 CW goat anti‐mouse IgG (1:20,000) (Li‐Cor Biosciences). After washing in 1X PBS, the filter was scanned using an Odyssey Infrared Imaging System (Li‐Cor Biosciences) for visualization of immunoreactive proteins. All steps were carried out at room temperature.

### RNA‐seq

Total kidney RNA isolation was performed as described (Kelly et al. 2013). RNA, 3 *μ*g, was fragmented with RNAase III, cDNA libraries constructed with SOLiD adaptors by reverse transcription (RT), and then sequenced by strand‐specific RNA‐seq of short 50 bp reads using the SOLiD 4 platform (Center for Medical Genomics at Indiana University School of Medicine) as described (Kelly et al. 2013). Sequence alignment to the UCSC rat genome database was performed using BFAST (Homer et al. 2009). Gene expression was calculated in the form of Reads per Kilobase Exon Model per million mapped reads (RPKM) (Mortazavi et al. 2008). To identify differentially expressed genes, we conducted Student's *t‐*test on the logarithmically transformed RPKM values, comparing DI versus LS and DS versus LS. Network analysis was performed using Metacore Software (GeneGo, Carlsbad, CA).

### Cellular iron levels

A colorimetric ferrozine‐based assay was used to quantify iron in cultured cells (Riemer et al. 2004). Cell lysates initially mixed with equal volumes of 1.4 mol/L HCl and 4.5% KMn0_4_, and then ferrozine iron‐detection reagent (6.5 mmol/L ferrozine, 6.5 mmol/L neocuproine, 2.5 mol/L ammonium acetate, and 1 mol/L ascorbic acid) followed by a 30‐min incubation and then absorbance measured at 550 nm. The concentration was calculated by comparing absorbance to a standard curve of FeCl_3_ standards.

### Animal use statement

The experiments were conducted in conformity with the “Guiding Principles for Research Involving Animals and Human Beings.” The investigations were approved by the Institutional Animal Care and Use Committee of Indiana University School of Medicine.

### Statistics

Results are expressed as means ± 1 standard error. Differences in renal and metabolic parameters were determined by one‐way analysis of variance (ANOVA) with subsequent *t*‐tests (when ANOVA indicated statistical significance, GraphPad Prism, LaJolla, CA). Bonferroni correction was used for multiple comparisons. The null hypothesis was rejected at *P* < 0.05.

## Results and Discussion

**Figure 2:**
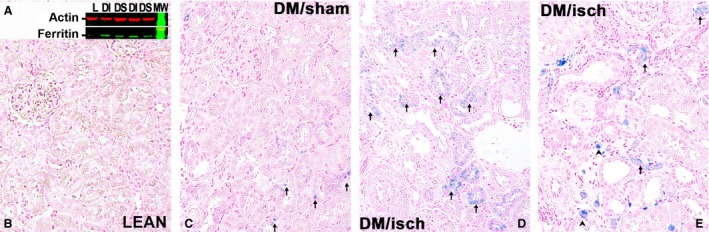
Renal ferritin on western blot and iron stain by Prussian Blue. Representative Western blot shows levels of the heavy chain of ferritin in kidneys from diabetic/obese rats, postischemia (DI), and in kidneys of sham‐operated obese/diabetic rats (DS). Ferritin was nearly undetectable in kidneys from normal sham‐operated lean rats (L), *n* = 4 for all, (A). Renal iron‐stained blue (arrows). Iron was also found in infiltrating leukocytes (arrowhead). Representative kidney sections from 28‐week‐old LS rats, Lean, (B); 28‐week‐old diabetic/obese, sham‐operated rats, DM/Sham, (C); 28‐week‐old, diabetic/obese ischemic rats, DM/isch, (D); and 36‐week‐old diabetic/obese ischemic rats, DM/isch, (E) are presented, *n* = 4 for all.

**Figure 3:**
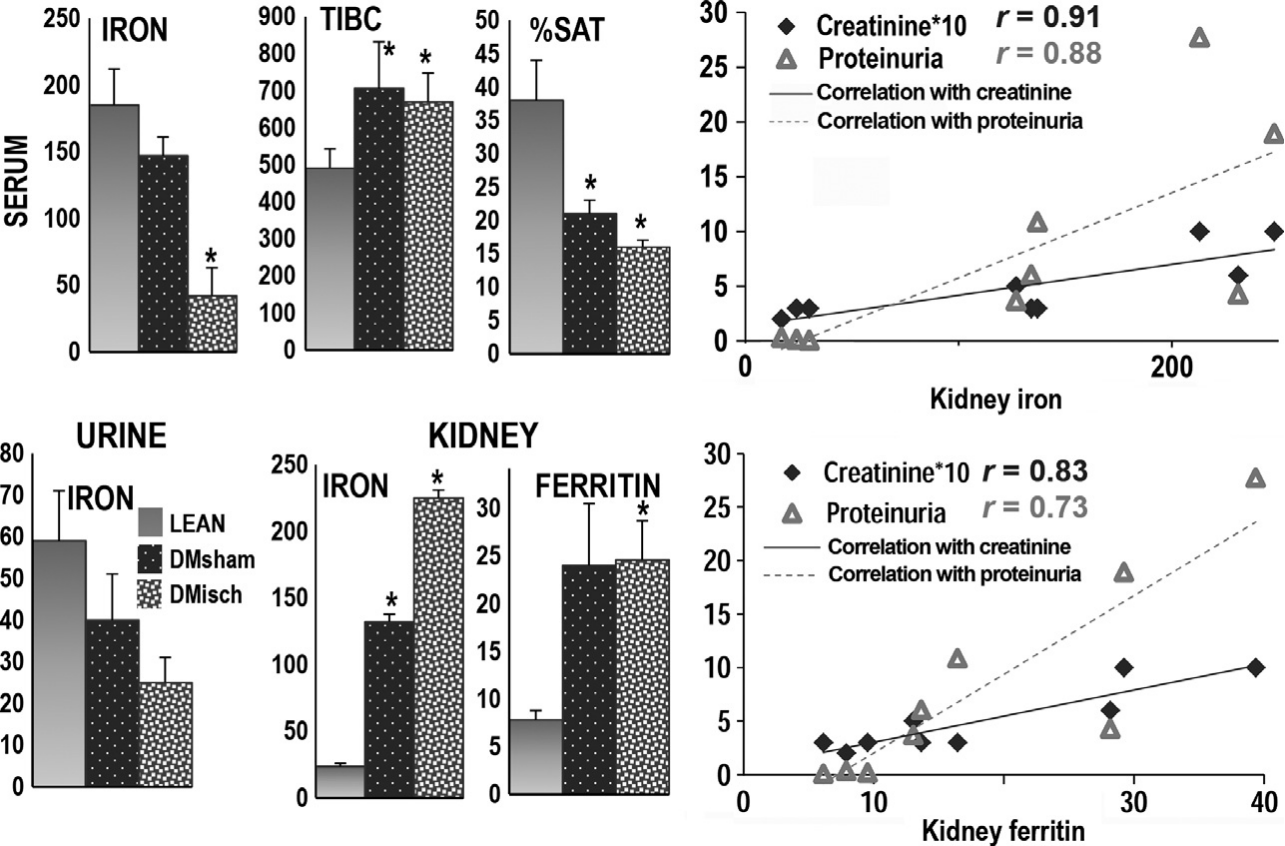
Iron levels in blood, urine and kidney. The left panels show that serum iron levels (*μ*g/100 mL) were lower in obese/diabetic ischemic rats, *n* = 11 (speckled, DMisch, DI;* P* < 0.002) and in obese diabetic sham‐operated rats, *n* = 7 (black/white dots, DMsham, or DS;* P* < 0.002) than in lean sham‐operated rats, *n* = 6 (gray, LEAN or LS). The clinical laboratory parameter total iron binding capacity (TIBC,* μ*g/100 mL), a surrogate measurement of transferrin, was higher in DMsham (*P* = 0.04) and DMisch (*P* < 0.004) than in LEAN. Hence, the percent saturation of serum TIBC (% SAT) by iron was lower in DMsham and DMisch rats (*P* < 0.002 for both) than in LEAN rats. Urine iron levels (*μ*g/24 h) were also lower in obese/diabetic ischemic rats (DMisch; *P* < 0.05) and obese/diabetic sham‐operated rats (DMsham) (NS) than in sham‐operated normal rats (LEAN). Levels of renal iron deposits (pixels of renal ferric ferrocyanide × 200 high power filed) were higher in DMisch (*P* = 2.9 × 10^−6^) and DMsham (*P* = 0.0005) than in LEAN rats. Renal heavy‐chain ferritin levels measured by Western blot were higher in DMisch (*P* < 0.05) and higher in DMsham (NS) than in LEAN. The ferritin heavy chain data were normalized to the corresponding actin band. The right panels show a positive correlation for serum creatinine levels (mg/100 × 10) and urine protein mg/mg creatinine with renal iron deposits (Top) and a positive correlation among serum creatinine and urine protein with renal ferritin levels (Bottom).

The abnormal metabolic and renal phenotypes in the ZS rats, subjects of this report, were described in detail in earlier articles (Kelly et al. 2013, 2015). There were three groups of ZS rats studied until 28 weeks of age: lean sham‐operated (LS), obese/diabetic sham‐operated (DS), and obese/diabetic subjected to a short period of bilateral renal ischemia (DI). There was a separate group of DI rats that was terminated at 36 weeks of age and included to demonstrate progressive renal iron retention (Figs. 2, 3). The previously published data (Kelly et al. 2013, 2015) showed that 28‐week‐old DS and DI rats were heavier than the LS controls. The blood glucose levels were also higher in DS and DI, respectively, when compared to LS controls. Creatinine clearance rates were depressed in DS and DI rats when compared to LS controls. Proteinuria, normal in LS, was increased in DS and DI. Renal histology showed cellular infiltrates that contained neutrophils and mononuclear cells in DI and less in DS. There was also more tubular damage plus interstitial and glomerular fibrosis, particularly in the DI rats (vs. LS). The original quantification and histology images have been shown in earlier publications (Kelly et al. 2013, 2015). Here we show the distribution of renal ferric ferrocyanide, or tissue iron, in LS, DS, and DI at 28 weeks of age, and also in DI at 36 weeks of age (Fig. 2).

Renal iron was found in some tubules of DS rats, but mainly in renal tubules of DI rats at 28 weeks of age and appeared more pronounced in DI rats at 36 weeks of age (also see (Nakhoul et al. 2009). In addition, levels of ferritin heavy chain were measured on western blots and found to be proportional to renal iron deposits, consistent with the finding that renal ferritin is a key source of total ferritin (Cohen et al. 2010), and that ferritin is also an important reservoir of iron (Fig. 3) (ten Kate et al. 1997).

Potential sources of accumulated renal iron in DS and DI were investigated by measuring serum and urine iron levels. We found that serum iron levels were severely depressed in DI, and marginally reduced in DS when compared to LS. We also measured total iron binding capacity (TIBC); a clinical laboratory indicator of the blood‐carrying iron capacity, and an indirect marker of transferrin levels. Total iron binding capacity was significantly higher in DS and DI, and thus TIBC percent saturation was lower in DS and DI, consistent with a state of iron deficiency in DS and DI when compared to LS, a result comparable to the human condition (ten Kate et al. 1997). Twenty‐four hour urine iron levels were proportional to serum iron levels, in that urinary iron levels in DS and DI were also lower than in LS. In marked contrast, renal iron and ferritin levels were higher in DS and DI. Accordingly, renal iron overload in diabetic rats does not appear to be caused by higher renal iron‐filtered loads. We also found that there was a positive correlation among serum creatinine and urinary protein with renal iron and also with renal ferritin levels, (Fig. 3).

**Figure 4:**
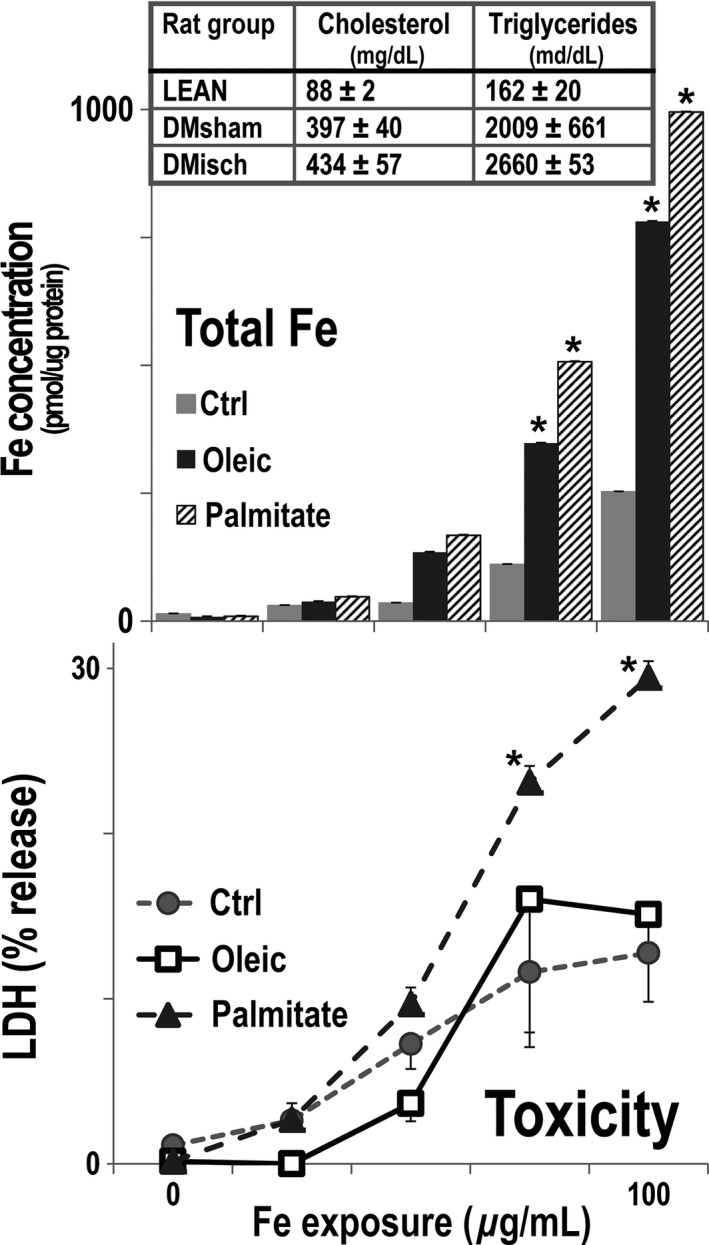
Serum lipids and cellular iron and toxicity in rat renal tubular cells. Serum cholesterol and triglycerides were significantly higher in DI (DMisch, *n* = 11), and DS (DMsham, *n* = 7) than in normal rats (LEAN,* n* = 6; *P* < 0.05 for all) (Top). Rat renal tubular cells were isolated from harvested rat renal tubules and cultured until confluent. The cells were maintained with 0.2 mmol/L oleate (dark gray), 0.2 mmol/L palmitate (cross hatch), or vehicle (light gray) for 48 h, *n* = 4. Sodium ferric gluconate, 0, 25, 50, 75 and 100 μg/mL was then added during the last 24 h of culture with the fatty acids. The two fatty acids increased the levels of cellular iron, *P* < 0.04 (Middle; *). The incremental addition of iron was toxic to renal tubular cells treated with vehicle (gray circle) and oleate (open squares). The addition of iron to palmitate‐ (gray triangles) treated cultures was more toxic than the other two groups, *P* < 0.007 (Bottom; *).

Tissue iron overload has been reported in humans with the metabolic syndrome, and in particular it has been strongly correlated with serum lipids, which are elevated in obese/diabetic ZS rats, (Fig. 4) (Howard et al. 1991), and also accumulate in kidneys of diabetic humans, mice (Kang et al. 2015), and ZS rats (Dominguez et al. 2007). Therefore, we tested whether the monounsaturated fatty acid oleic acid, and the saturated fatty acid palmitate, increased iron in cultured renal tubular cells from kidneys of normal lean male ZS rats. After an initial 24 h of exposure to the fatty acids, iron was added to the culture media for 24 more hours in the continued presence of the fatty acids. The two fatty acids caused substantial increments in renal tubule iron accumulation, (Fig. 4). Palmitate addition was more cytotoxic, as manifested by tubular LDH release, wheras oleic acid or iron had lower and comparable effects on tubular LDH release.

**Figure 5:**
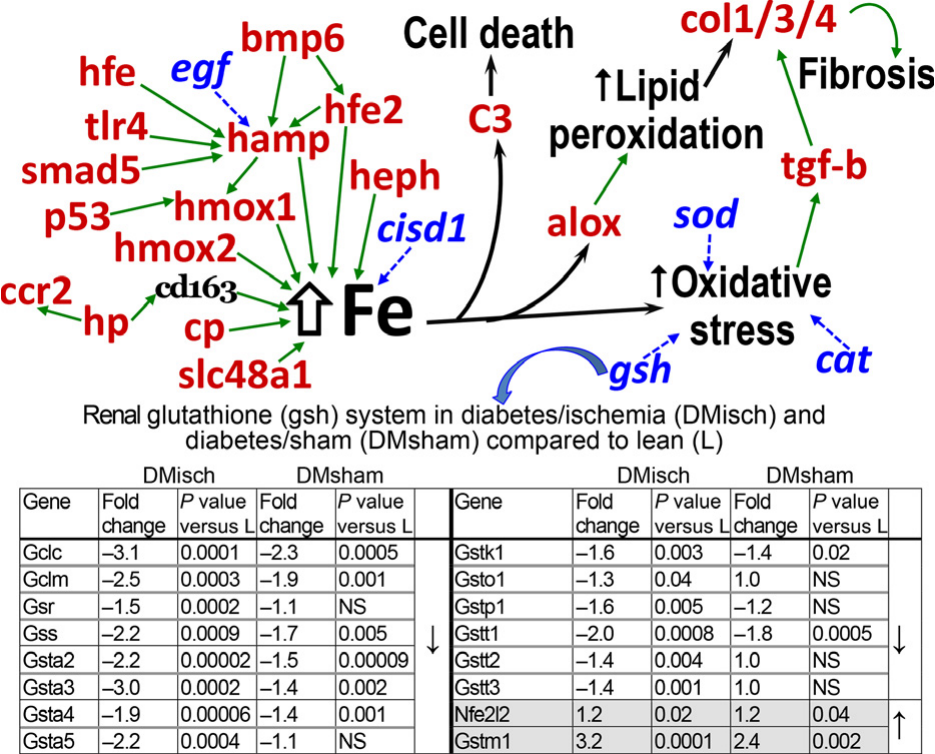
Renal iron metabolism gene pathways in diabetic nephropathy. The network on the left side of iron accumulation (Fe) contains upregulated (red), inhibited (blue, italics) (*n* = 4 for all three groups, differences were significant, *P* < 0.05) or unchanged transcripts (black). Green arrows show positive interactions. The network placed upregulated renal Hamp (Hepcidin) in a key position to promote iron overload, along with other upregulated genes: Hfe2 (Hemochromatosis type 2), Hmox1/2 (Heme oxygenase 1 and 2), Hp (Haptoglobin), Cp (Ceruloplasmin), and Slc48a1 (Heme transporter 1). On the right side of Fe it is shown that renal C3 component activation (Kelly et al. 2015) and Alox (Arachidonate lipoxygenase) are activated by iron loads. C3 activation leads to cell death via its derived C3b component (Kelly et al. 2015), and Alox is involved in lipid peroxidation. Iron itself induces oxidant stress and renal fibrosis via upregulated TGFbeta. Oxidant stress is compounded by the inhibition of the renal genes encoding the antioxidants SOD (superoxide dismutase), CAT (catalase), and of the components of glutathione synthesis (embedded table).

Renal transcripts encoding interacting proteins involved in iron metabolism were assembled in a gene network (Fig. 5). These transcripts were differentially expressed in both conditions, DS and DI, when compared to control transcripts (LS). The center piece of the network is accumulated renal iron. The upper left corner of the network shows increased levels of the iron‐sensitive genes Bmp6 and its coreceptor Hfe2 (Hjv, Valenti et al. 2012; Gkouvatsos et al. 2014). Bmp6 is stimulated in iron overload (Parrow and Fleming 2014), and it is shown interacting positively with the renal Hamp (hepcidin) gene (McDonald et al. 2014). This effect might be initiated by renal inflammation and is thought to limit iron efflux from cells, promoting intracellular iron retention (Babitt et al. 2006). These local interactions are ostensibly intrarenal, whereas systemic hepcidin activation might have a different and unrelated outcome (Parrow and Fleming 2014). The activated renal Hfe (hemochromatosis) gene directly regulates Hamp providing additional stimulatory input (Babitt et al. 2007). Furthermore, suppressed renal epidermal growth factor (EGF) in DI/DS is less likely to restrain activated hepcidin (Gulec et al. 2014). The network also points to a positive interaction between the genes coding for hepcidin and heme oxygenase 1 (Hmox1) (Latour et al. 2014), which in turn is acted on by renal proapoptotic p53 (Kartikasari et al. 2009) also stimulated in DI/DS (Kelly et al. 2013). It is anticipated that renal iron loads in DI/DS might be increased by the degradation of renal heme, secondary to stimulated Hmox1 (Nam and Sabapathy 2011). Another potential source of renal heme, and iron, is from free hemoglobin, which, when complexed with stimulated renal haptoglobin (Hp), is internalized via renal CD163 prior to lysosomal degradation (Evstatiev and Gasche 2012). The figure alludes to the potential role of two upregulated renal genes that facilitate iron transfer into tissues, the multi‐copper oxidases Cp (ceruloplasmin) and Heph (hephaestin) (Madsen et al. 2001). The figure also includes upregulated Slc48a1 (heme responsive gene‐1), which promotes iron overload by delivering heme from the endosome to the cytosol (Vashchenko and MacGillivray 2013). On the left side of the figure, it is shown that DI/DS‐induced suppression of Cisd1, which encodes the mitochondrial protein Mitoneet that potentially causes renal mitochondrial iron overload as shown by others (Khan and Quigley 2013).

Figure 5 shows the elevated renal iron catalyzes the lipid peroxidation reactions of lipoxygenases (Alox) (Percival 1991; Kusminski et al. 2012), which can stimulate renal fibrosis via generated lipid peroxides (Neau et al. 2014). Renal ischemia causes iron overload (Paller and Hedlund 1988; Dominguez et al. 2008), and also activates renal C3 component (Kelly et al. 2015), with the latter secondary to the former (Shah et al. 2011). Iron can also catalyze the formation of reactive oxygen intermediates, largely responsible for lipid peroxidation and cell damage (Fig. 5) (de Vries et al. 2004). The renal prooxidant stress in rats with DI (Dominguez et al. 2007) is further compounded by suppression of critical antioxidant transcript systems, including catalase (down −1.7; *P* < 0.0002), superoxide dismutase (down −1.8; *P* < 0.0002), and the enzymes that comprise the depleted renal glutathione antioxidant system of obese/diabetic rats (Neau et al. 2014). For example, in the embedded table in Figure 5 is shown that renal genes encoding critical components of the glutathione synthetic pathway are generally depressed. It is noteworthy that Nfe2l2 (Nrf2), a major regulator of cell response to oxidative stress is slightly increased, but this reaction seems to be inadequate (Ishii and Mann 2014). Finally, renal decline in DI is characterized by fibrosis (Dominguez et al. 2007), and reactive oxidant species can promote fibrogenesis in part by the action of TGF beta 1 on the upregulated collagen genes 1, 3, and 4 (Liu and Gaston Pravia 2010) (Fig. 5).

Diabetic nephropathy is a complex and deadly complication, and its progression is mediated by a host of factors: proinflammatory, proapoptotic, antiproliferative, and profibrotic (Kelly et al. 2013, 2015). We suggest that renal damage might be further accelerated by iron overload, with significant prooxidant and proinflammatory consequences (Papanikolaou and Pantopoulos 2005).

## Conflict of Interest

None declared.
